# HECW2 promotes the progression and chemoresistance of colorectal cancer via AKT/mTOR signaling activation by mediating the ubiquitin-proteasome degradation of lamin B1

**DOI:** 10.7150/jca.87545

**Published:** 2023-09-04

**Authors:** Fang Li, Li Wang, Yujue Wang, Hui Shen, Qianrui Kou, Changjun Shen, Xiangrong Xu, Yunqing Zhang, Jing Zhang

**Affiliations:** 1 Medical School of Yan'an University, Yan'an 716000, China.; 2 Yan'an University Affiliated Hospital, Yan'an 716000, China.; 3 Yan'an People's Hospital, Yan'an 716000, China.

**Keywords:** colorectal cancer, progression, chemoresistance, HECW2, lamin B1, AKT/mTOR signaling pathway

## Abstract

Colorectal cancer (CRC) is among the most common malignancies worldwide. Although a recent study has shown that E3 ubiquitin ligases play a major role in regulating the occurrence and development of CRC, there are few reports on the role of the E3 ubiquitin ligase HECW2(HECT, C2 and WW domain containing E3 ubiquitin protein ligase 2) in CRC progression and chemoresistance. We found that HECW2 is highly expressed in CRC tissues. HECW2 knockdown inhibits CRC progression and chemoresistance, whereas HECW2 overexpression has the opposite effect. Mechanistically, HECW2 activates the AKT/mTOR signaling pathway by mediating the ubiquitin-proteasome degradation of lamin B1, thereby promoting CRC progression and chemoresistance. Our findings suggest that HECW2 may be a promising novel therapeutic target for CRC treatment.

## Introduction

Colorectal cancer (CRC) is one of the most malignant tumors in clinic and is a leading cause of tumor-related mortality worldwide 1. Despite the improvements in diagnostic methods and therapeutic strategies, CRC prognosis is still poor. Therefore, it is important to further elucidate the molecular mechanisms underlying the progression and chemoresistance of CRC.

HECW2 (HECT, C2 and WW domain containing E3 ubiquitin protein ligase 2), which is also known as NEDL2, is a member of the E3 ubiquitin ligase NEDD4 family. Previous studies on the function of HECW2 have mainly focused on the neurodevelopmental disorders and dysgnosia 2, 3. Research has shown that HECW2 may be involved in regulating the development of various tumors, including CRC. For instance, HECW2 enhances the transcriptional activity of the tumor protein p73 (TP73) 4. In HEK293T cells, HECW2 mediates ubiquitin-proteasome degradation of proliferating cell nuclear antigen (PCNA) and lamin B1, and studies have shown that PCNA and lamin B1 play major roles in CRC 5-8. CircRNA HECW2 has been reported to positively regulate LPS-induced EMT (epithelial-mesenchymal transition), a biological process that plays a critical role in the development of CRC 9, 10. Moreover, the protein expression levels of HECW2 are significantly up-regulated in colon cancer tissues 11. Additionally, E3 ligases play a key role in chemoresistance, which results in CRC-treatment failure 12. TRIM47 promotes CRC chemoresistance to 5-FU by facilitating the ubiquitination and degradation of SMAD4 13. FBXW7 targets CRY2 through proteasomal degradation, thereby suppressing the oxaliplatin chemoresistance of CRC cells 14. However, the role of HECW2 in CRC progression and chemoresistance remains unclear.

The AKT/mTOR signaling pathway plays a vital role in the regulation of proliferation, migration, invasion and metabolism of CRC cells 15, 16. The aberrant activation of AKT/mTOR signaling pathway is considered to be a classical oncogenic factor of CRC 17, 18. Notably, ubiquitination is a key step in AKT activation 19. And AKT is a candidate ubiquitination substrate for HECW2. However, whether HECW2 activates AKT in an ubiquitination-dependent manner and regulates CRC progression and chemoresistance via AKT/mTOR signaling pathway remains unclear.

Lamin B1, one of the two B-type lamin proteins, is an important component of the nuclear lamina 20. Studies have shown that lamin B1 regulates CRC development 8, 21, 22. For example, β-Asarone promotes senescence in CRC cells by inducing lamin B1 expression, thereby inhibiting CRC progression 8. In addition, Overexpression of lamin B1 increases the 5-FU sensitivity of colon cancer cells LoVo 6. However, the role of lamin B1 ubiquitination in the progression and chemoresistance of CRC has not been reported so far.

In the present study, HECW2 was found to be highly expressed in CRC tissues and cells. HECW2 knockdown inhibited the progression and chemoresistance of CRC, whereas the opposite effect was observed in HECW2-overexpressing CRC cells. More importantly, HECW2 activated the AKT/mTOR signaling pathway by mediating ubiquitin-proteasome degradation of lamin B1, thereby promoting CRC progression and chemoresistance. Collectively, these findings provides new insights into the mechanisms underlying CRC progression and chemoresistance. Thus, HECW2 may be a promising therapeutic target for CRC.

## Materials and methods

### Cell culture and major reagents

The human CRC cell lines were obtained from a cell bank at the Medical Research and Experimental Center of Yan'an University. HCT116, HT-29 and RKO cells were cultured at 37°C, 5% CO2 in RPMI 1640 medium, Mc-Coy's 5A and DMEM medium with 10% FBS (GIBCO), respectively. SC79 (specific AKT activator) and MK-2206 (specific AKT inhibitor) were obtained from Selleck (S7863) and Beyotime (SF2712), respectively. 5-Fluorouracil and irinotecan hydrochloride were purchased from Sigma (F6627) and Selleck (S5026), respectively.

### CRC tissues collection

CRC tissues and the paired normal tissues were obtained from CRC patients who were treated in Yan'an People's Hospital or Yan'an University Affiliated Hospital from September 2020 to September 2022. All samples were diagnosed and classified by the department of pathology. Inclusion criteria: all patients were diagnosed as primary colorectal cancer by postoperative pathological examination, and there was a clear pathological diagnosis classification. Exclusion criteria: patients with other types of primary cancer (such as cervical cancer) were excluded. This study was approved by the Ethics Committee of Yan 'an University.

### Cell transfection

The HECW2 siRNAs and lamin B1 siRNAs were purchased from GenePharm (Shanghai, China); HECW2 plasmid and lamin B1 plasmid were obtained from Genechem (Shanghai, China). The HECW2 siRNAs, lamin B1 siRNAs and HECW2 plasmid were transfected according to standard protocols by using jetPRIME^®^ transfection reagent (Polyplus, 114-15). The lamin B1 plasmid was transfected according to standard protocols by using Lipofectamine^TM^ 3000 Reagent (Thermo Fisher Scientific, L3000015). HECW2 siRNA, forward (5′-3′): GGGAGAAGAUCCAAUUUAUTT, reverse (5′-3′): AUAAAUUGGAUCUUCUCCCTT; lamin B1 siRNA, forward (5′-3′): GCAGACUUACCAUCGCAAATT, reverse (5′-3′): UUUGGCAUG GUAAG UCUGCTT.

### qRT-PCR

Total RNA was extracted from CRC cells using TRIzol reagent (ambion, 15596018) and converted into cDNA using EasyScript One-step gDNA Removal and cDNA Synthesis SuperMix (Transgene, AE311-03). Quantitative real-time PCR was performed using Perfect Start Green qPCR Super Mix (Transgene, TG-AQ601-02). The sequences of the primers were as follows: HECW2, 5′-CCAGAGTTCTTCACCGTGCT-3′ (forward), 5′-CCACAAAGAATGCCTTGCCC-3′ (reverse); CDK4, 5′-ATGGCTACCTCTCGATATGAGC-3′ (forward), 5′-CATTGGGGACTCTCACACTCT-3′ (reverse); Cyclin D1, 5′-ATCTACACCGACAACTCCATC-3′ (forward), 5′-TGTTCTCCTCCGCCTCTG-3′ (reverse); MMP2, 5′-TGACTTTCTTGGATCGGGTCG-3′ (forward), 5′-AAGCACCACATCAGATGACTG-3′ (reverse); MMP9, 5′-CCCGGACCAAGGATACAGTTT-3′ (forward), 5′-GTGCCGGATGCCATTCAC-3′ (reverse); β-Actin, 5′-CCAACCGCGAGAAGATGA-3′ (forward), 5′-CCAGAGGCGTACAGGGATAG-3′ (reverse).

### Western blotting

Cells were lysed with RIPA lysis buffer and protein concentrations were assessed by BCA assay kit (Pioneer Biotechnology, PP-01). Western blotting was performed according to standard protocols. The following antibodies were used: anti-HECW2 (Abcam, ab92711, 1:1000), anti-MMP9 (Proteintech, 10375-2-AP, 1:2000), anti-MMP2 (Proteintech, 10373-2-AP, 1:2000), anti-CDK4 (Proteintech, 11026-1-AP, 1:3000), anti-Cyclin D1 (Proteintech, 26939-1-AP, 1:3000), anti-lamin B1 (Proteintech, 12987-1-AP, 1:5000), anti-phospho-AKT (Ser473) (Proteintech, 66444-1-Ig, 1:1000), anti-AKT (Proteintech, 60203-2-Ig, 1:2000), anti-phospho-mTOR (Ser2448) (Proteintech, 67778-1-Ig, 1:2000), anti-mTOR (Proteintech, 66888-1-Ig, 1:4000), anti-β-actin (Transgene, HC201-02, 1:4000).

### CCK8 assay

Transfected CRC cells were seeded into 96-well plates (3000cells/well). And CCK8 reagent (10μL/well) was added to each well at 24h, 48h, 72h and 96h after seeding. Then the absorbance was measured at 450nm after 2h incubation at 37°C.

### Cell cycle assay

CRC cells were transfected in six-well plates for 48h. Then, the cells were fixed with 70% ethanol overnight at -20°C, then washed with PBS, stained with PI dye solution (100 μg/mL) and RNaseA (500 μg/mL). The DNA content was detected by flow cytometry.

### Wound healing assay

Cells were seeded into six-well plates, then transfected after 24h. When cells were cultured to nearly 90% confluence, a scratch was made in the cell layer using a 10µL sterile pipette tip. Then cells were washed with PBS, and incubated in the medium with 1% FBS and the images of the wound closure were captured at 0h, 24h, 48h and 72h.

### Transwell assay

Cells were seeded in the medium with 1% FBS in the upper chamber, and 600μL medium with 10% FBS was added to the lower chamber. After 48h incubation, cells were fixed with 4% paraformaldehyde. Cells adhered to the underside of the membrane were stained with 0.1% crystal violet and the images were captured under microscope. For the invasion assay, the upper chamber was covered by the matrigel (Abwbio, 0827045) before seeding cells. Next steps are the same as the migration experiment.

### Co-immunoprecipitation (IP) assay

Whole-cell extracts were preincubated with protein A and G beads (P2018, Beyotime), then incubated with IgG (Beyotime, A7016) and lamin B1 antibody overnight at 4℃, respectively. The beads were washed three times with Tris-HCL buffered saline, and the immunoprecipitation complexes were subjected to SDS-PAGE.

### Ubiquitination assay

CRC cells were transfected in six-well plates for 72h. Then the cells were lysed with mammalian cell lysis reagent (Pioneer Biotechnology, K0301), followed by overnight incubation at 4℃ with lamin B1 antibody (Proteintech, 12987-1-AP). The cell extracts were then incubated with protein A and G beads for 3h. The beads were washed three times with Tris-HCL buffered saline, and the immunoprecipitation complexes were subjected to SDS-PAGE.

### Statistical analysis

SPSS 22.0 software was used for statistical analysis. The data were calculated from at least three independent experiments and presented as the mean±SD. A two-group comparison was determined by Student's t test. Comparison of more than two groups was conducted by one-way ANOVA test. *P*<0.05 was considered statistically significant.

## Results

### HECW2 is overexpressed in CRC

**Figure 1:**
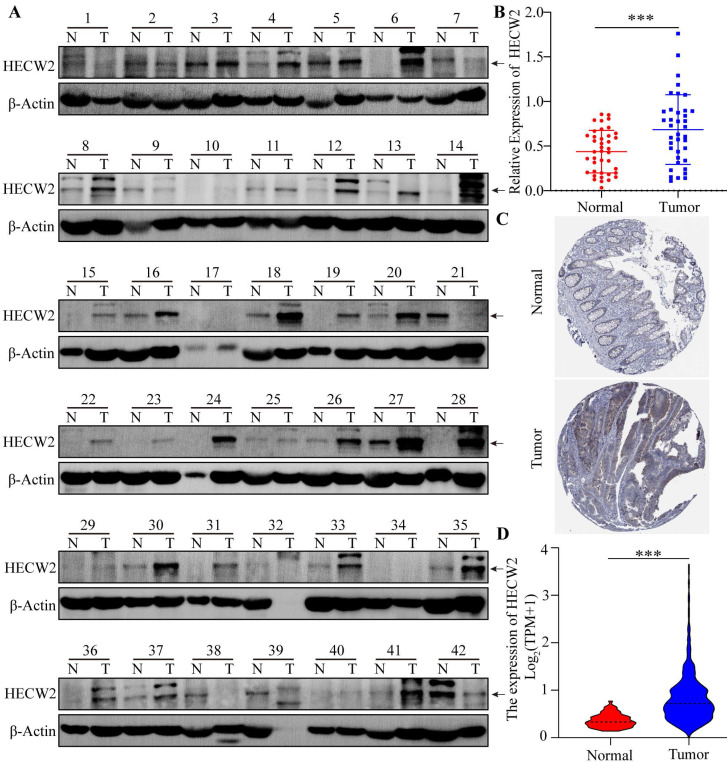
HECW2 is overexpressed in CRC tissues. **(A-B)** The protein expression levels of HECW2 in CRC tissues from 42 patients was assessed by western blotting. N: Paired normal tissues, T: Tumor tissues(CRC tissues). N(Normal)=38, N(Tumor)=38. **(C)** The protein expression levels of HECW2 in CRC tissues and normal tissues from HPA database. **(D)** The relative mRNA expression levels of HECW2 in CRC tissues and normal tissues from TCGA database. N(Normal)=51, N(Tumor)=647. (mean±SD, ***P< 0.001).

HECW2 was highly expressed in CRC tissues as shown by western blotting results(Fig. 1A-B). Moreover, bioinformatics analysis showed that the protein and mRNA expression levels of HECW2 were significantly higher in CRC tissues than that in normal tissues, as shown in HPA and TCGA samples (Fig. 1C-D). As shown in the K-M curves, CRC patients with high expression levels of HECW2 had a lower overall survival than those with low HECW2 expression levels, indicating that HECW2 is positively correlated with the poor prognosis of CRC (Fig. S1A).

### HECW2 promotes the proliferation and cell-cycle progression of CRC cells

With the exception of RKO cells, the protein expression levels of HECW2 in CRC cell lines were significantly higher than those in NCM-460 cells (Fig. S1B). HCT116, HT-29 and RKO cells were selected for further experiments to elucidate the role of HECW2 in CRC progression. The efficiency of knockdown and overexpression of HECW2 was validated using western blotting and qRT-PCR (Fig. S1C-E).

**Figure 2:**
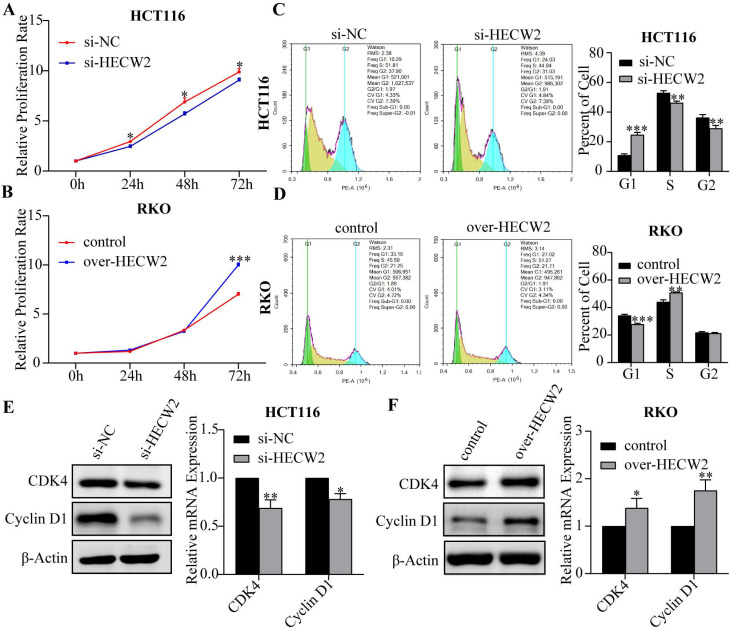
HECW2 promotes the proliferation and cell-cycle progression of CRC cells. **(A-B)** The effect of HECW2 on the proliferation of CRC cells. **(C-D)** The effect of HECW2 on cell cycle progression of CRC cells. **(E-F)** The effect of HECW2 on the expression levels of marker proteins of G1/S phase transition. (mean±SD, N=3, *P< 0.05, **P< 0.01, ***P< 0.001).

As shown in Fig. 2A and Fig. S1F, HECW2 knockdown significantly inhibited the proliferation of HCT116 and HT-29 cells, whereas a contrary effect is observed in HECW2-overexpressing RKO cells (Fig. 2B). And knockdown of HECW2 led to a significant increase in the number of CRC cells in the G0/G1 phase and a decrease of those in the S phase, suggesting that the down-regulation of HECW2 blocked the cell cycle of CRC cells at the G0/G1 phase (Fig. 2C and Fig. S1G). On the contrary, HECW2 overexpression promoted the cell cycle progression in CRC cells (Fig. 2D). Additionally, the expression levels of CDK4 and Cyclin D1 in CRC cells decreased with HECW2 down-regulation (Fig. 2E), and increased with HECW2 overexpression (Fig. 2F).

### HECW2 promotes the migration and invasion of CRC cells

**Figure 3:**
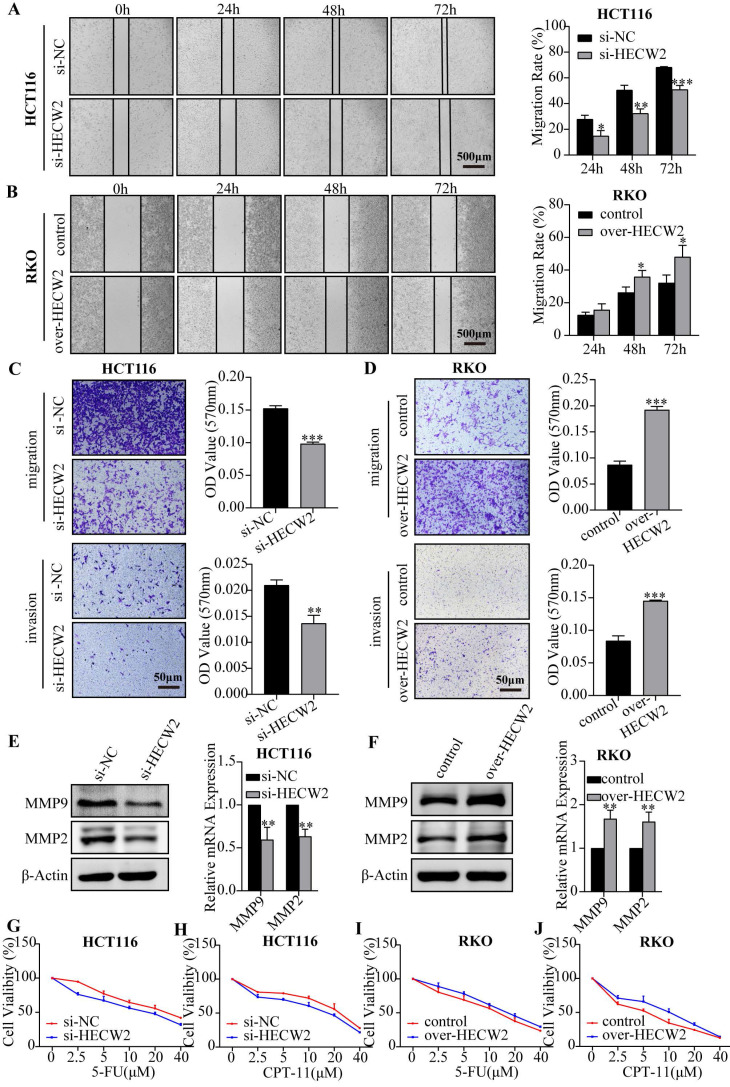
HECW2 promotes the migration, invasion and chemoresistance of CRC cells. **(A-B)** Wound healing assays were performed to evaluate the effect of HECW2 on the migration of CRC cells. **(C-D)** The effect of HECW2 on the migration and invasion in CRC cells was assessed by transwell assays. **(E-F)** The effect of HECW2 on the protein and mRNA expression levels of MMP2 and MMP9 in CRC cells. **(G-J)** The effect of HECW2 on the chemoresistance of CRC cells. (mean±SD, N=3, *P< 0.05, **P< 0.01, ***P< 0.001).

Subsequently, we determined whether HECW2 plays a role in the migration and invasion of CRC cells. The wound healing assay showed that HECW2 knockdown significantly inhibited wound closure in CRC cells compared to that in controls (Fig. 3A and Fig. S1H). Conversely, HECW2 overexpression promoted wound closure in CRC cells (Fig. 3B). A transwell assay demonstrated that migration and invasion rates were significantly lower in HECW2-knockdown and higher in HECW2-overexpressing CRC cells (Fig. 3C-D). Moreover, HECW2 knockdown resulted in significantly lower expression levels of MMP9 and MMP2 in CRC cells at the protein and mRNA levels (Fig. 3E). The converse was true for HECW2-overexpressing CRC cells (Fig. 3F).

### HECW2 promotes the chemoresistance of CRC cells

Numerous studies have shown that E3 ubiquitin ligases are involved in regulating CRC chemoresistance. To determine whether HECW2 affects the chemoresistance of CRC cells, we evaluated the response of HECW2-knockdown and HECW2-overexpressing CRC cells to 5-FU and irinotecan hydrochloride (CPT-11). HECW2 knockdown sensitized CRC cells to 5-FU and CPT-11, whereas HECW2 overexpression desensitized them, which indicated that HECW2 may promote the chemoresistance in CRC cells (Fig. 3G-J and Fig. S1I-J).

### HECW2 activates AKT/mTOR signaling pathway in an ubiquitination- independent manner

**Figure 4:**
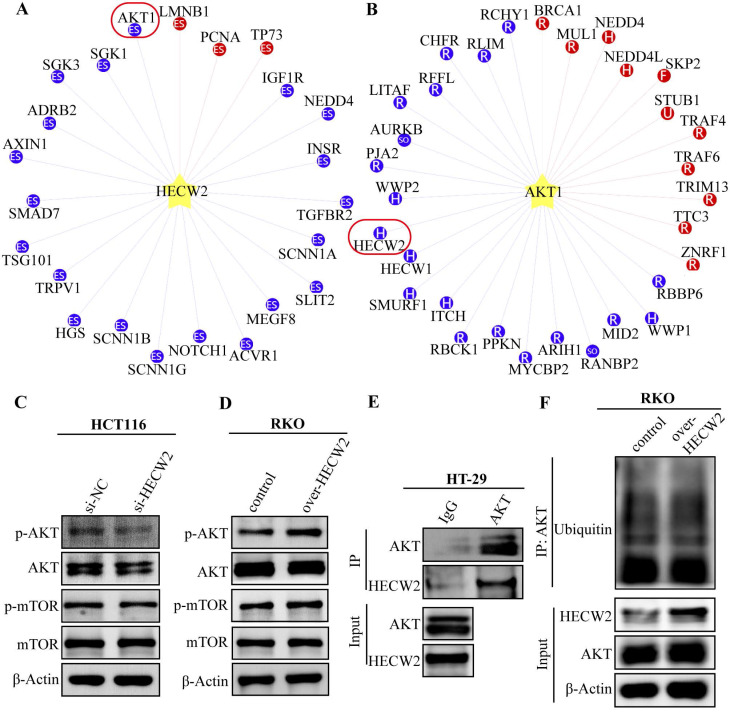
HECW2 activates AKT in an ubiquitin-independent manner. **(A)** AKT was a candidate ubiquitination substrate for HECW2. **(B)** HECW2 was a candidate E3 ubiquitin ligase for AKT. **(C)** The effect of HECW2 knockdown on the activation of AKT and mTOR. **(D)** The effect of HECW2 overexpression on the activation of AKT and mTOR. **(E)** The interaction between HECW2 and AKT was detected by the Co-IP assay.** (F)** The effect of HECW2 overexpression on the ubiquitination levels of AKT.

To clarify the molecular mechanisms underlying the HECW2 regulation of CRC progression and chemoresistance, we used the UbiBrowser database (http:// ubibrowser.bio-it.cn) and found that AKT is a candidate ubiquitination substrate for HECW2 (Fig. 4A). HECW2 is also predicted as a candidate E3 ubiquitin ligase for AKT (Fig. 4B). AKT/mTOR signaling is a classic pathway involved in CRC development, and ubiquitination is a key steps in AKT activation. We speculated that HECW2 may activate AKT through ubiquitination, then mediating CRC progression and chemoresistance via AKT/mTOR signaling pathway.

As expected, HECW2 knockdown resulted in significantly reduced phosphorylation levels of AKT and mTOR in CRC cells but had no significant effect on the protein expression levels of total AKT and mTOR (Fig. 4C). Conversely, a marked increase in the phosphorylation levels of AKT and mTOR was observed in HECW2-overexpressing CRC cells (Fig. 4D). These results indicated that HECW2 was able to activate the AKT/mTOR signaling pathway. Notably, HECW2 could interact with AKT in CRC cells, which was confirmed by a Co-IP assay (Fig. 4E). However, we observed that HECW2 overexpression had no obvious effect on AKT ubiquitination levels (Fig. 4F). Thus, HECW2 may activate AKT in a ubiquitination-independent manner and facilitate CRC progression and chemoresistance via AKT/mTOR signaling pathway.

### HECW2 activates AKT/mTOR signaling through ubiquitin-proteosome degradation of lamin B1, thereby promoting CRC progression and chemoresistance

**Figure 5:**
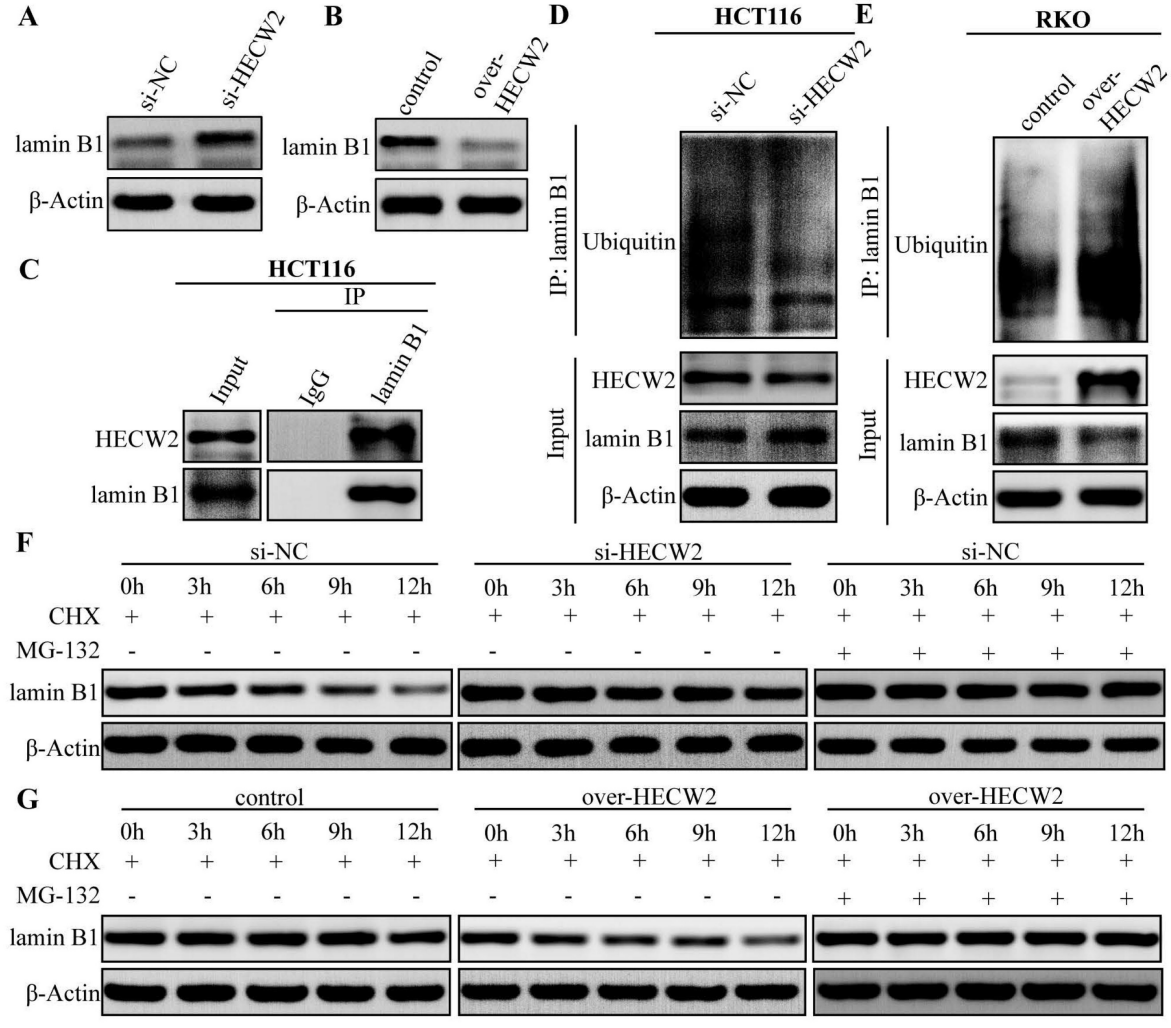
HECW2 mediates the ubiquitin-proteasome degradation of lamin B1 in CRC cells. **(A-B)** The effect of HECW2 knockdown or overexpression on the protein expression levels of lamin B1 in CRC cells. **(C)** The interaction between HECW2 and lamin B1 was detected by the Co-IP assay. **(D-E)** The effect of HECW2 knockdown or overexpression on the ubiquitination levels of lamin B1. **(F-G)** MG-132 could reverse the proteasomal degradation of lamin B1 mediated by HECW2.

Since HECW2 activation of the AKT/mTOR signaling pathway was not dependent on ubiquitination, its underlying mechanism needed to be further elucidated. Given that lamin B1 is an upstream regulator of the AKT/mTOR signaling pathway and a candidate substrate for HECW2, we explored whether lamin B1 is involved in the activation of AKT/mTOR signaling pathway mediated by HECW2. As shown in Fig. 5A-B, HECW2 knockdown resulted in significantly higher expression levels of lamin B1 in CRC cells, whereas the opposite results were observed in HECW2-overexpressing CRC cells. HECW2 could interact with lamin B1 in CRC cells as evidenced by the Co-IP assay (Fig. 5C). Furthermore, HECW2 knockdown markedly decreased the ubiquitination levels of lamin B1 (Fig. 5D). On the contrary, overexpression of HECW2 significantly increased the ubiquitination levels of lamin B1 (Fig. 5E). Compared to the controls, lamin B1 (in the presence of cycloheximide) exhibited higher and lower protein stability in CRC cells knockdown and overexpressing HECW2, respectively (Fig. 5F-G). Moreover, the proteasome inhibitor MG-132 could reverse the ubiquitin-proteasome degradation of lamin B1 mediated by HECW2 (Fig. 5F-G). Collectively, these findings suggested that HECW2 mediates the ubiquitin-proteasome degradation of lamin B1.

**Figure 6:**
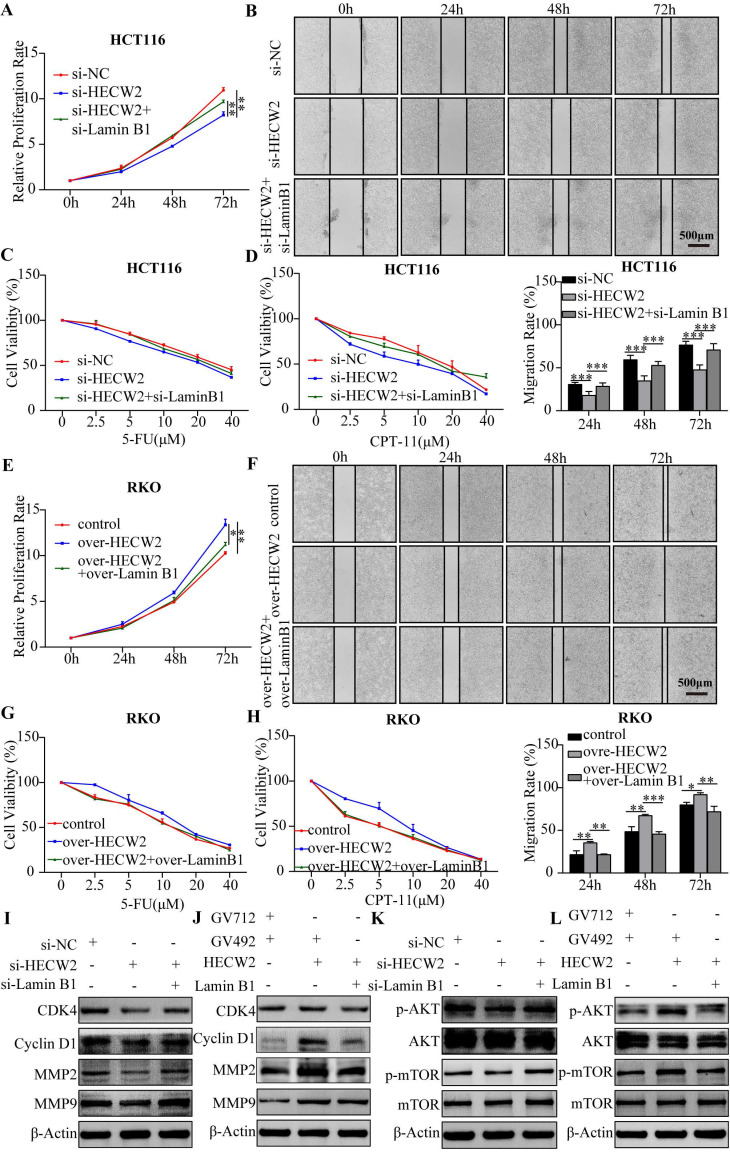
HECW2 mediates the ubiquitin-proteasome degradation of lamin B1, thereby promoting CRC progression and chemoresistance. **(A-B)** Downregulating lamin B1 could reverse the inhibiting effect of HECW2 knockdown on the proliferation and migration of CRC cells. **(C-D)** Downregulating lamin B1 could rescue the inhibiting effect of HECW2 knockdown on the chemoresistance of CRC cells. **(E-F)** Upregulating lamin B1 could reverse the promoting effect of HECW2 overexpression on the proliferation and migration of CRC cells. **(G-H)** Upregulating lamin B1 could attenuate the effect of HECW2 overexpression on the chemoresistance of CRC cells. **(I-J)** Western blotting was performed to assess the effect of knockdown and overexpression of lamin B1 on the expression levels of marker proteins of cell cycle, migration and invasion in CRC cells. **(K-L)** The effect of knockdown and overexpression of lamin B1 on the activation of AKT and mTOR was detected by western blotting. (mean±SD, N=3, *P< 0.05, **P< 0.01, ***P< 0.001).

Furthermore, we explored whether HECW2 promotes CRC progression and chemoresistance by mediating the ubiquitin-proteasome degradation of lamin B1. As shown in Fig. 6A-D, lamin B1 knockdown negated the inhibitory effects of HECW2 knockdown on the proliferation, migration, and chemoresistance of CRC cells. Accordingly, overexpression of lamin B1 reversed the promoting effects on the proliferation, migration, and chemoresistance of HECW2 overexpression in CRC cells (Fig. 6E-H). These findings were validated by western blotting (Fig. 6I-J). Furthermore, knockdown and overexpression of lamin B1 reversed the effects of HECW2 knockdown and overexpression on the activation of AKT/mTOR signaling, respectively (Fig. 6K-L). Thus, it may be stated that HECW2 promotes CRC progression and chemoresistance by mediating the ubiquitin-proteasome degradation of lamin B1.

**Figure 7:**
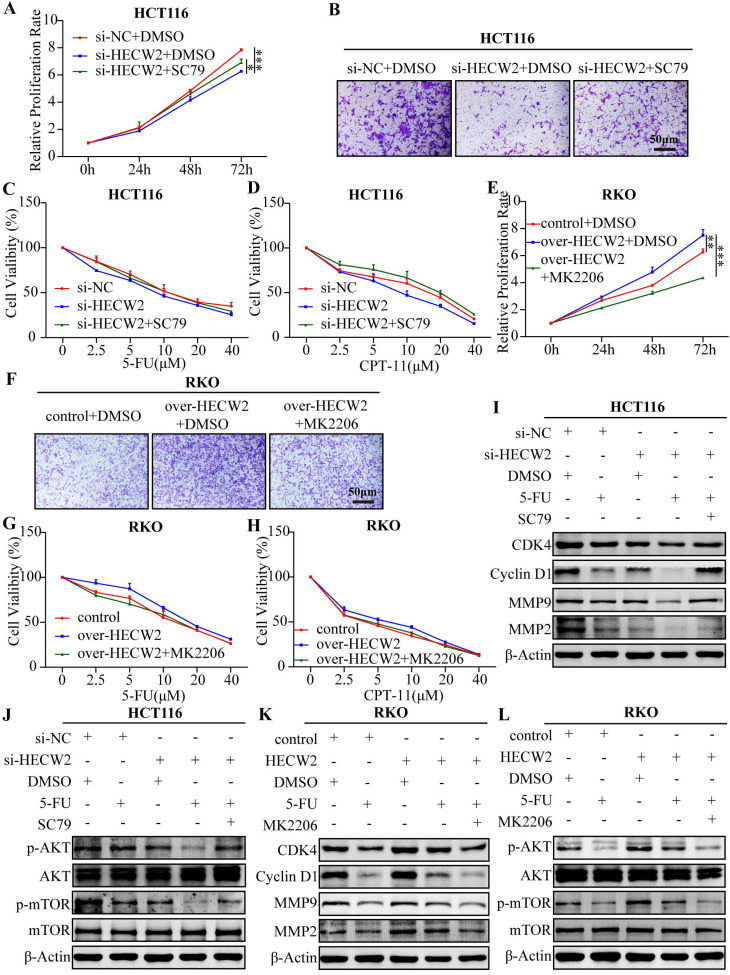
SC79 and MK-2206 reverse the effect of HECW2 knockdown and overexpression on CRC progression and chemoresistance. **(A-B)** SC79 could reverse the effect of HECW2 knockdown on the proliferation and migration of CRC cells. **(C-D)** SC79 could reverse the effect of HECW2 knockdown on the chemoresistance of CRC cells. **(E-F)** MK-2206 could reverse the effect of HECW2 overexpression on the proliferation and migration in CRC cells. **(G-H)** MK-2206 could reverse the effect of HECW2 overexpression on the chemoresistance of CRC cells. **(I-J)** The effect of SC79 on the expression levels of marker proteins of cell cycle, migration and invasion, as well as the activation of AKT and mTOR in CRC cells. **(K-L)** The effect of MK-2206 on the expression levels of marker proteins of cell cycle, migration and invasion, as well as the activation of AKT and mTOR in CRC cells. (mean±SD, N=3, *P< 0.05, **P< 0.01, ***P< 0.001).

As shown in Fig. 7A-D, SC79 (a specific AKT activator) invalidated the inhibitory effects of HECW2 knockdown on the proliferation, migration, and chemoresistance of CRC cells. Contrastingly, MK2206 (a specific AKT inhibitor) attenuated the promoting effects of HECW2 overexpression on the proliferation, migration, and chemoresistance of CRC cells (Fig. 7E-H). These results were confirmed by western blotting (Fig. 7I-L). In conclusion, HECW2 activates the AKT/mTOR signaling pathway by mediating the ubiquitin-proteasome degradation of lamin B1, thereby promoting CRC progression and chemoresistance.

## Discussion

**Figure 8:**
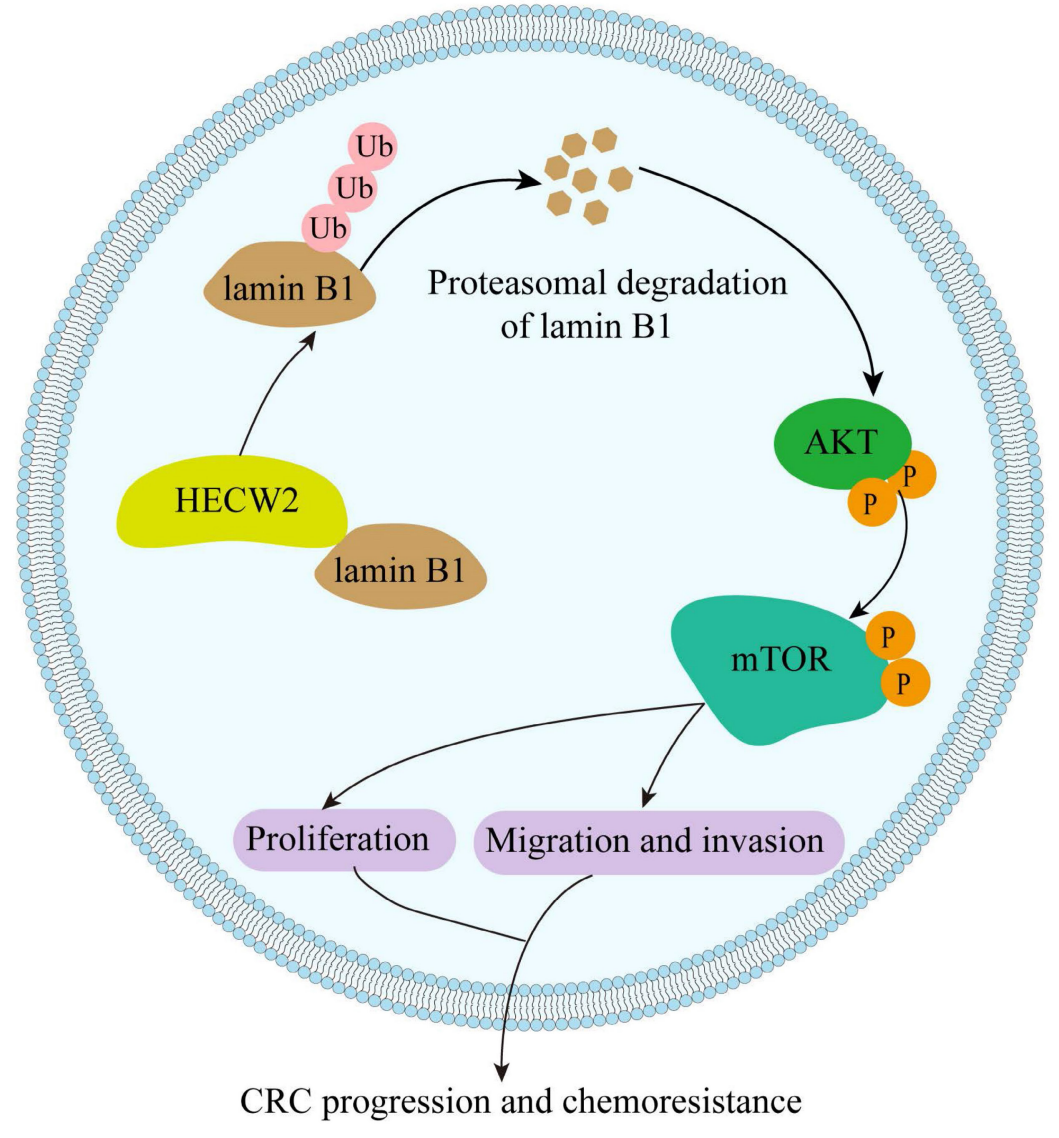
A working model of HECW2 in regulating CRC progression and chemoresistance.

In the present study, we elucidated the role of the E3 ubiquitin ligase HECW2 in the progression and chemoresistance of CRC for the first time. We found that HECW2 activated the AKT/mTOR signaling pathway by mediating the ubiquitin-proteasome degradation of lamin B1, thereby promoting CRC progression and chemoresistance (Fig. 8). This demonstrated the potential of HECW2 as a novel therapeutic target for CRC.

Most studies on HECW2 have focused on neurodevelopmental disorders and dysgnosia 2, 3. To our knowledge, this study elucidate the role of HECW2 in CRC progression and chemoresistance for the first time. Our results demonstrated that HECW2 is significantly overexpressed in CRC. Similarly, the protein expression levels of HECW2 in colon cancer tissues are markedly higher than those in normal tissues 11. Recent studies have shown that HECW2 may be able to mediate the proteasomal degradation of karyopherin subunit alpha 1 (KPNA1) and MORC family CW-type zinc finger 4 (MORC4) in CRC cells. Given that KPNA1 and MORC4 could promote CRC progression, it is speculated that HECW2 may act as a tumor suppressor in CRC 23, 24. In contrast, our results showed that HECW2 overexpression promotes CRC progression and chemoresistance, whereas HECW2 knockdown inhibits it. Furthermore, evidences shows that HECW2 may also play an important role in regulating the development of other tumors. HECW2 is significantly overexpressed in cervical cancer and may act as a potential oncogenic factor for human papillomavirus 11, 25. HECW2 is also considered as a new candidate hub gene for the targeted therapy of congenital giant melanocytic nevi 26.

In the present study, we found that HECW2 activates AKT, thereby promoting CRC progression and chemoresistance via AKT/mTOR signaling pathway. Studies have shown that AKT interacts with HECW2 in HEK293T cells 27. In line with this, our results demonstrated that AKT could interact with HECW2 in CRC cells. Interestingly, there were no obvious implications for the ubiquitination levels of AKT during knockdown or overexpression of HECW2 in CRC cells, although AKT was predicted to be a candidate ubiquitination substrate for HECW2. It indicated HECW2 activates AKT in an ubiquitination-independent manner. And HECW2 may activate the AKT/mTOR signaling pathway by regulating the upstream factors of AKT via its ubiquitination activity. We found that HECW2 could mediate the ubiquitin-proteasome degradation of lamin B1. Consistent with this, it has been reported that lamin B1 is an upstream regulator of the AKT/mTOR signaling pathway and is proteasomal degradated by HECW2 in HEK293T cells 5, 28. Moreover, lamin B1 plays a vital role in regulating CRC progression and chemoresistance 6, 8.

In conclusion, HECW2 activates the AKT/mTOR signaling pathway by mediating the ubiquitin-proteasome degradation of lamin B1, thereby promoting CRC progression and chemoresistance. Thus, inhibition of HECW2 may be a novel efficient therapeutic approach for CRC.

## Supplementary Material

Supplementary figure.
