# Supplementary material for: HECW2 promotes the progression and chemoresistance of colorectal cancer via AKT/mTOR signaling activation by mediating the ubiquitin-proteasome degradation of lamin B1
Source: J Cancer. 2023 Sep 4;14(15):2820–32. doi: 10.7150/jca.87545 (PMC10539569; doi:10.7150/jca.87545)
Supplement: Supplementary file 1 — Supplementary figure. [file jcav14p2820s1.pdf]

Fig. S1 HECW2 knockdown inhibits the proliferation, cell-cycle progression, migration as well as chemoresistance of HT-29 cells. **(A)** K-M curves of CRC patients based on HECW2 expression levels. **(B)** The protein expression levels of HECW2 in CRC cell lines. **(C-E)** The efficiency of HECW2 knockdown and overexpression in CRC cells was validated by western blotting and qRT-PCR. **(F)** The effect of HECW2 knockdown on the proliferation of HT-29 cells. **(G)** The effect of HECW2 knockdown on the cell-cycle progression of HT-29 cells. **(H)** The effect of HECW2 knockdown on the migration of HT-29 cells. **(I-J)** The effect of HECW2 knockdown on the chemoresistance of HT-29 cells.(mean ± SD, N=3, *$P < 0.05$, **$P < 0.01$, ***$P < 0.001$)

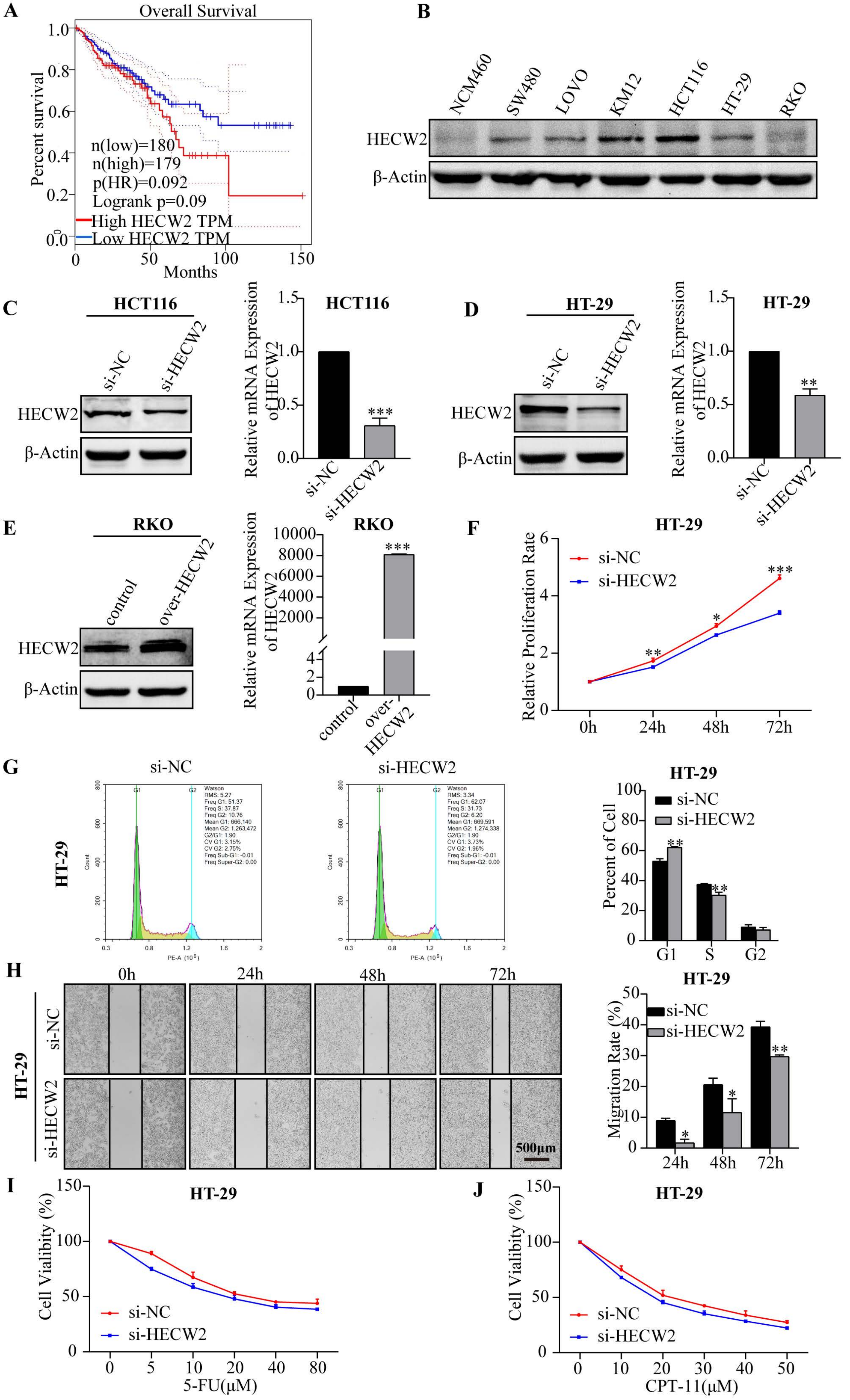
A
Overall Survival
Percent survival
n(low)=180
n(high)=179
p(HR)=0.092
Logrank p=0.09
High HECW2 TPM
Low HECW2 TPM
Months
B
NCM460
SW480
LOVO
KM12
HCT116
HT-29
RKO
HECW2
β-Actin
C
HCT116
si-NC
si-HECW2
HECW2
β-Actin
Relative mRNA Expression of HECW2
***
D
HT-29
**
E
RKO
control
over-HECW2
***
F
HT-29
Relative Proliferation Rate
0h
24h
48h
72h
G
si-NC
si-HECW2
Percent of Cell
G1
S
G2
H
0h
24h
48h
72h
500μm
Migration Rate (%)
I
HT-29
Cell Viability (%)
5-FU(μM)
J
CPT-11(μM)
